# Nanoscale Imaging of Synaptic Connections with Expansion Microscopy

**DOI:** 10.15190/d.2019.14

**Published:** 2019-09-30

**Authors:** Brendan Gallagher, Yongxin Zhao

**Affiliations:** Department of Biological Sciences, Carnegie Mellon University, Pittsburgh, PA, USA

**Keywords:** Expansion microscopy, synapse, dopaminergic neurons, nanoscale imaging.

## Abstract

Biologists have long looked for ways to circumvent the physical diffraction limit of light and have developed many strategies to accomplish this. While many techniques employed to image sub-diffraction-limit structures rely on sophisticated equipment and computational methods, expansion microscopy (ExM) is unique in that it provides increase in resolution by physically expanding the sample embedded in a water-swellable hydrogel. ExM has rapidly grown in prevalence, owing to its ease of use and economic nature – all necessary reagents are commercially available, and samples may be imaged in large volume on conventional fluorescence microscopes. Here, we demonstrate the power of expansion microscopy on imaging synaptic connections onto a dopaminergic neuron, in the mouse substantia nigra pars compacta, with nanoscale resolution.

Discovered by Ernst Abbe in 1873, the physical diffraction limit of light dictates the ultimate resolution provided by a far-field optical microscope, which cannot be better than approximately half of the wavelength of the light. Such limitation precludes observation of structures and molecular organization in cells and tissues with nanoscopic details using optical microscopy. In the past decade, there have been many advancements that seek to circumvent the optical diffraction limit, such as 4pi microscopy^1^, Stimulated Illumination Microscopy (SIM)^2^, Stimulated Emission Depletion (STED) microscopy^3^, and Stochastic Optical Reconstruction Microscopy (STORM)^4^, etc. Each of these methods have their own benefits and drawbacks: SIM and 4pi microscopy are easy to perform but provide relatively moderate resolution increase when compared with other super-resolution techniques^3,5^, while STED and STORM provide a marked increase in resolution, but they require sophisticated equipment and expertise^5^, limiting their applications in most laboratories with limited personnel and budget. In addition, it is challenging to use these techniques for tissue imaging, especially at larger volumes, due to limited working distance in axial direction, significant background and light scattering^5^.

Expansion microscopy (ExM) allows for visualization of cellular structures below the physical diffraction limit of light. It works by cross-linking monomers that constitute a water-swellable polymer to biomolecules in a stained tissue sample, followed by *in situ *hydrogel formation^6^, mechanical homogenization and isotropic expansion in water. After expansion, the sample can be typically as large as 100 folds of its original volume. Novel composition of water-swellable hydrogel may allow even larger expansion of the specimen^7^. Since more than 99% of space is filled with water, tissue expansion renders the whole sample transparent, which facilitates large volume analyses with nanoscale resolution^6,8^. Expanded samples may be imaged on a standard fluorescent microscope without need for expensive or custom equipment^8^. Depending on the optical setup and expansion factor, lateral resolution of up to 40 nm can be attained in a fully expanded sample without need for additional hardware upgrades^8^. The expansion of a tissue sample adds at most two days to an immunostaining protocol, with minimal active operation time. Thus, ExM is an inexpensive and effective method for imaging nanoscopic structures that can be employed in nearly any biological laboratory.

Here, we demonstrate the ability of ExM to resolve synapses in the mouse brain, specifically synapses on to the soma of a neuron in the substantia nigra *pars compacta *(SNc). The SNc is a midbrain nucleus mostly comprised of dopaminergic neurons^9^. A major projection target of these neurons is to the striatum, and the death of these neurons are a hallmark of Parkinson’s Disease. Paraformaldehyde-fixed mouse brain tissue was immunostained against the dopaminergic neuron marker tyrosine hydroxylase (TH), synaptophysin, a pre-synaptic vesicle marker, and post-synaptic marker PSD95 (**Figure 1A**). Prior to expansion, puncta indicative of a synaptic pair can be seen, though often these labels appear overlapping. The dopaminergic neuron can be clearly seen, and tyrosine hydroxylase is observed uniformly throughout. A closer look at the surface of the neuron reveals two synapses onto the neuron. Synaptic markers appear as partially overlapping solid puncta laying directly on the TH (**Figure 1C**). The sample was then expanded 7.2× in each dimension, more readily revealing synaptic pairs and cellular TH localization (**Figure 1B**). On the soma, synaptophysin and PSD95 are clearly separated, revealing the synaptic cleft (**Figure 1D**). We envision that with further development, ExM will become an indispensable imaging tool for studying nanoscopic structure and organization of synaptic components in the brain tissues.

**Figure 1:**
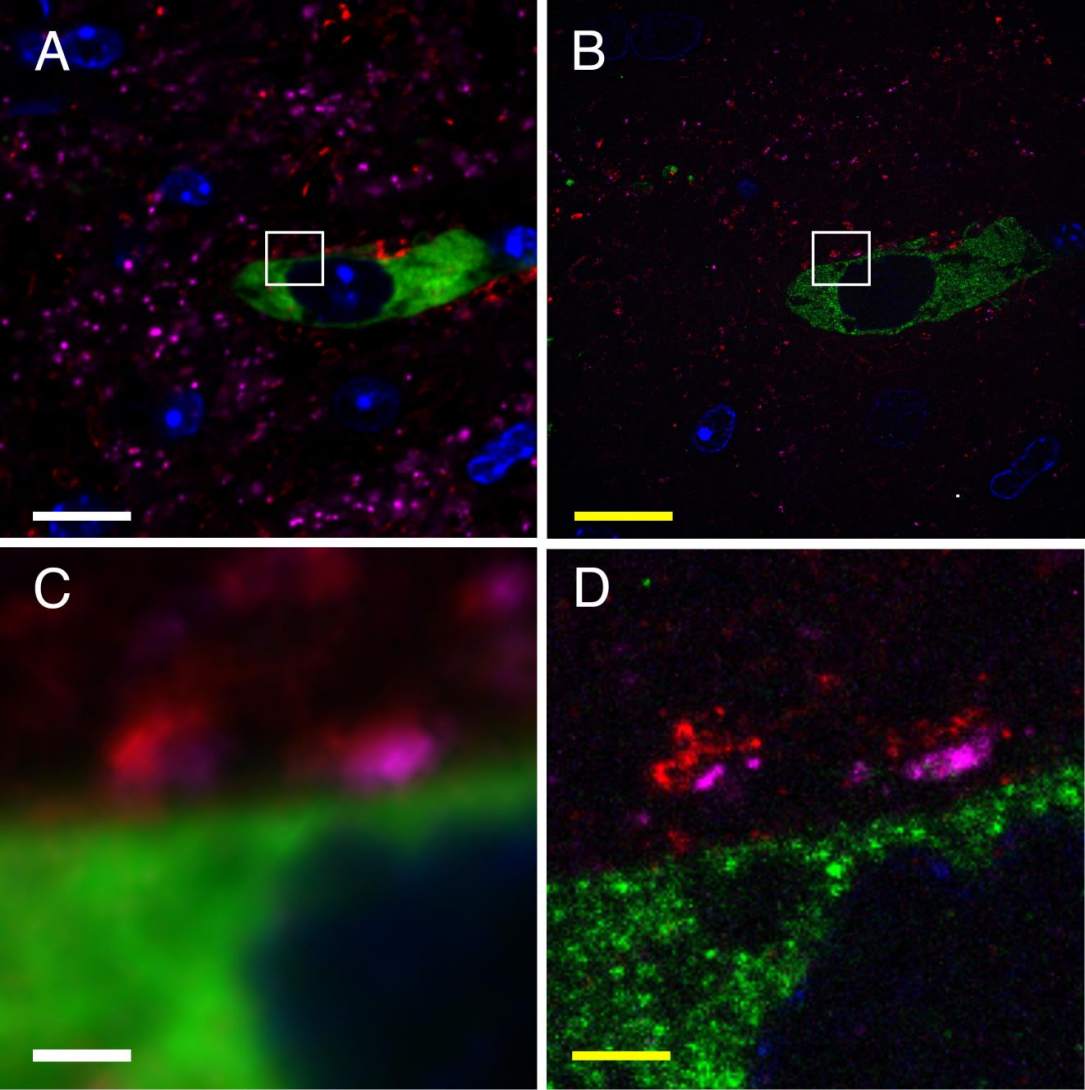
**A.** Unexpanded and **B.** expanded images of a dopaminergic neuron in the mouse substantia nigra pars compacta. Sagittal mouse brain sections were stained with DAPI (blue) and labeled for tyrosine hydroxylase (green), synaptophysin (red), and PSD95 (magenta). **C. **and **D.** Zoomed-in images of boxed regions in **A.** and **B.** showing individual synapses, respectively. Images taken under a Nikon Ti2 Eclipse inverted fluorescence microscope equipped with CSU-W1 spinning disk confocal module using a long working distance water immersion 40× objective. Scale bars (biological scale): **A.** and **B.**, 10 μm; **C.** and **D.**, 0.5 μm; Expansion factor: 7.2× in pure water.
